# Psychological stress dysfunction in women with premenstrual syndrome

**DOI:** 10.1016/j.heliyon.2024.e40233

**Published:** 2024-11-12

**Authors:** Qing Liu, Yuhang Lin, Wenjuan Zhang

**Affiliations:** a College of Education, Zhejiang University of Technology, Hangzhou, China; b Mental Health Education Center, Xidian University, Xi'an, China

**Keywords:** Menstrual cycle, Premenstrual syndrome, Stress reactivity, Coping, Dysfunction

## Abstract

Premenstrual syndrome (PMS) encompasses a range of emotional, physiological, and behavioral symptoms that occur during the luteal phase of the menstrual cycle (MC) and resolve with the onset of menstruation. These symptoms, which can include fatigue, physical pain, anxiety, irritability, and depression, significantly affect women's daily lives and overall well-being. In severe cases, PMS can progress to premenstrual dysphoric disorder (PMDD), profoundly impairing quality of life. Despite its prevalence, the neural mechanisms underlying PMS—particularly those related to stress—are not fully understood.This review aims to explore the complex interactions between PMS and stress, with a focus on the hormonal pathways involved. We propose that abnormal stress coping styles and stress reactivity patterns, collectively referred to as stress dysfunction, are crucial factors influencing women's vulnerability to PMS. We examine the relationship between PMS and stress from four perspectives: (1) PMS shares neuroendocrine metabolic circuits based on hormonal fluctuations with stress reactivity systems; (2) there is comorbidity between PMS and stress-related disorders; (3) PMS itself may act as a stressor, potentially creating a negative feedback loop that exacerbates symptoms; and (4) biofeedback training used for stress disorders may be effective in treating PMS. By providing a detailed analysis of stress-related hormonal changes and their effects on PMS, this review offers new insights into the physiological processes underlying PMS. Understanding these interactions may inform the development of targeted interventions and improve the quality of life for women affected by PMS.

## 1 Introduction

Women experience hormonal changes at key life cycle inflection points (such as first menstruation/menarche, pregnancy, childbirth, perimenopause, and menopause), involving increased or decreased levels of fertility steroids such as estrogen and progesterone [1]. In the case of hormonal transition or flux, women experience slight mood swings [2]. Women are generally more likely to experience negative symptoms such as fatigue, physical pain, anxiety, irritability, and depression during menstruation, which affect their daily lives [3]. In severe cases, extreme behavior, such as suicide, may occur [4]. For some women, normal hormone conduction can be associated with exacerbation of mental illness, such as bipolar disorder and major depression [5]. Menstrual cycle-related issues are closely related to women's overall health. Therefore, it is necessary to conduct further research on women's menstrual cycle (MC) issues.

Premenstrual syndrome (PMS) is a group of emotional, physiological, and behavioral symptoms related to the MC. These symptoms generally appear in the premenstrual phase or luteal phase of the MC and disappear at the end of the MC [6]. The premenstrual dysphoric disorder (PMDD) as the most severe premenstrual disorder, debilitates and severely interferes with quality of life [7]. Researchers believe that during childbearing age, women generally experience 400–500 MCs, and that premenstrual symptoms peak 4–7 days before the start of the MC, so women with PMS will spend 4–10 years in a state of impaired physical function or psychological well-being [8]. PMS often starts after menarche, but women generally do not want to seek treatment to solve PMS problems until after the age of 30, indicating that by the time they seek treatment they would have experienced premenstrual discomfort for more than ten years. Therefore, we should consider PMS as a major health problem for women, as Campagne and Campagne put it: “Compared with other forms or conditions of disease, more women and their families will suffer from premenstrual symptoms” [9]. In this sense, PMS is a social issue worthy of attention.

“This review aims to provide a comprehensive analysis of the complex interactions between premenstrual syndrome (PMS) and stress, with a focus on the neuroendocrine mechanisms and stress reactivity that link the two. Unlike previous reviews, such as the one by Modzelewski et al. [10], which primarily explores treatment methods for PMS and PMDD, or the review by Takeda et al. [11], which emphasizes the diagnosis and treatment challenges in PMS, our review highlights the bidirectional relationship between stress and PMS and examines how stress-related hormonal fluctuations contribute to PMS symptoms.Additionally, compared to Gurvich et al. [12], which discusses cognitive changes across the menstrual cycle in PMS and PMDD patients, our review extends the discussion by incorporating recent findings on stress reactivity. By organizing the current evidence and proposing potential intervention strategies like biofeedback, this review offers novel insights into PMS as a stress-related disorder.”

In 2012, the fifth edition of the Diagnostic and Statistical Manual of Mental Disorders V (DSM-V) included PMDD as a separate diagnostic category for mood disorders [13]. Due to the different definitions of PMS and PMDD used in different studies, the epidemiological data on PMS and PMDD has varied greatly. Some studies even pointed out that 80–90 % of women will feel more or less premenstrual discomfort during childbearing age [14]. There are also studies on large samples of community groups, which confirmed that the annual incidence rate of PMS is 5.8 %, and the lifetime incidence rate is as high as 7.4 % [15]. Finally, it seems that PMS and PMDD widely exist, not only in Western countries represented by the United States, but across cultures [[16], [17], [18], [19]]. In the field of epidemiology, research on PMS is necessary.

There is a high degree of comorbidity between PMS and other mental disorders. This means that women with PMS are more likely to suffer from other mental disorders. The lifetime comorbidity between PMS and other mood disorders (women who suffer from PMS suffer from another mood disorder at a certain stage of the life cycle) is estimated to be 30–70 % [[20], [21], [22], [23]]. Specifically, the comorbidity rate between PMS and interpersonal sensitivity is 44.6 %, the comorbidity rate between PMS and paranoia is 42.8 %, and the comorbidity rate between PMS and phobic anxiety is 46.4 % [20]. Among women with PMS, 31 % met the criteria for a mood disorder [21], 25 % met the criteria for generalized anxiety disorder (GAD), and 25 % reported having panic disorder at the same time [22]. Women with PMS are also at higher risk for perimenopausal depression and postpartum depression [20]. In addition, there are also comorbidities between PMS and stress-related disorders [[24], [25], [26], [27], [28], [29]]. Accordingly, the study of PMS is complicated because its etiology is uncertain, leading to difficulties in its diagnosis and treatment [6]. The establishment of accurate diagnostic criteria and effective treatment measures is related to its etiology. In order to facilitate diagnosis and treatment, some researchers believe that PMS is a psycho-neural endocrine disorder that has stress as its main cause [27]. Some strategies for intervention with PMS are mainly aimed at reducing or controlling the stress of women with PMS and improving their mood to suppress their physical discomfort [28,29]. Thus, the relationship of PMS and stress should be outlined systematically. While the review is a narrative, rather than a systematic review, it is still important and helpful to provide both clear aims and an outline of literature search strategies.

## 2 Stress reactivity, coping styles, sensibility and vulnerability

Stress exists everywhere in daily life and has an impact on individual happiness, health, and cognition. Our brains must continuously adapt to stressful environments. Therefore, stress can be defined as the process of inducing organisms to make adaptive adjustments in line with environmental requirements [30]. Our bodies respond to almost all events or challenges by releasing chemical transmitters. For example, releasing catecholamines can increase heart rate and blood pressure and help us cope with certain situations. The chronic release and accumulation of catecholamines and the associated increase of the individual's heart rate and blood pressure, will affect the cardiovascular system in the long term. Eventually, individuals may experience stroke and heart disease [31].“These physiological reactions are particularly relevant in women with PMS/PMDD, as the body's stress response mechanisms are heightened during the luteal phase, exacerbating symptoms like anxiety, mood swings, and irritability due to hormonal fluctuations [32].”

**Fig. 1:**
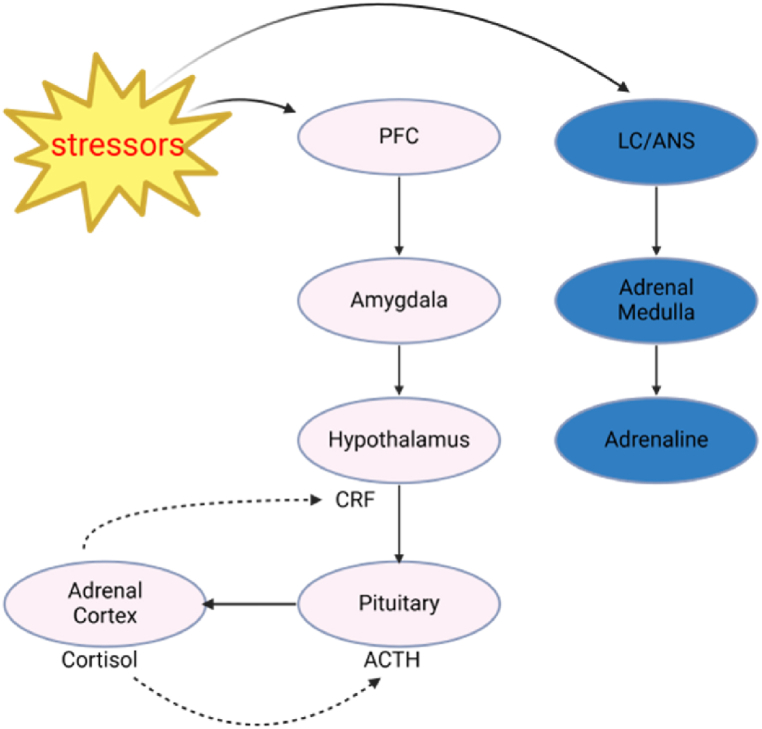
The psychological, physiological and endocrinological systems are activated in response to stress.

Our bodies respond to stress physiologically, and this physiological response is amplified or reduced by stress regulation. Our brain perceives a source of stress through the interaction between the prefrontal lobe and amygdala. The prefrontal lobe first recognizes the existence of a stressor, and then the amygdala makes an emotional evaluation of this stressor [33].“The sympathetic nervous system (SNS) including pathways involving the locus coeruleus (LC) and autonomic nervous system (ANS) responds to stress by increasing heart rate, blood pressure, and breathing, while inhibiting non-essential functions like digestion. This response, along with HPA axis dysregulation, exacerbates emotional disturbances in PMS [34].” The most rapid response to stress is the activation of the SNS system, which is regulated by catecholamines, adrenal hormones, and noradrenaline.“The HPA axis triggers the release of glucocorticoids like cortisol, which enhances physiological arousal in response to stress. This hormonal dysregulation, particularly within the HPA axis, plays a key role in the escalation of PMS symptoms [35].“The activation of ACTH eventually causes the release of corticosteroids in the blood vessels, one of which is cortisol, an important corticosteroid hormone (Fig. 1).

In short, the central nervous system (CNS) determines whether there is a threat and directs the body to respond. The amygdala helps the brain respond to threats and stores memories of past threatening experiences. The hypothalamus and brainstem activate the HPA and SNS systems [36]. Insufficient pressure response reduces the HPA axis's response to stress, resulting in decreased sympathetic nerve activity and a natural decrease in cortisol secretion. A negative feedback system is located in the pituitary gland, where the hypothalamus and hippocampus function to regulate the secretion of cortisol [37]. The role of the HPA axis as an integrated component in the human physiological response to stress is subject to the top-down regulation of the frontal cerebral cortex and the amygdala. Regulation of the physiological response to stress can be achieved by measuring HPA axis activation and byproducts of the SNS [38].

According to the biological principle of stress, under stressful conditions, the secretion of glucocorticoids first affects the individual's SNS activity, and then the HPA axis [39]. Our previous research showed that when the SNS is restored and glucocorticoids continue to be secreted, the impact of stress on individual cognition has been eliminated [40]. Autonomic indicators change in seconds and can return to baseline levels within minutes; however, it takes 20–40 min for cortisol and other hormone responses to reach the peak, and it takes about 1 h to return to baseline [41]. Based on hormone-regulated stress response neural plasticity, from the dynamic changes of stress measurement, sympathetic nerve indicators are more flexible and feasible [42,43]. How people cope with stress affects physiological reactivity to stress and its impact on general health.

Survival depends on the ability to successfully respond to threats. Effective stress reactivity is passed on through generations according to the principles of natural selection. Those who do not successfully react to threats are unlikely to live to childbearing age. The successful stress reactivity of humans is generally labeled as “fight-or-flight” reactivity and has always been regarded as a representative of key mechanisms in the survival processes [44]. “Fight or flight” reactivity was originally proposed by Walter Cannon. It was identified as the first physiological arousal of the SNS that stimulated the adrenal medulla and produced a cascade of hormones that caused the release of catecholamines, especially stress hormones such as noradrenaline and adrenaline, to flow into the blood vessels [45]. In addition to the physiological arousal reactivity, “fight or flight” is also used as a metaphor for human behavioral reactivity to stress. The decision to fight or flee (flight) when physiological arousal occurs depends on the nature of the stress experienced. If the organism faces a threat or a predator, and then considers it a viable option to defeat the opponent, then it may fight. Conversely, organisms are more likely to choose to flee when the threat is considered sufficiently powerful [46].

Although “fight or flight” reactivity can indicate the main physiological and behavioral reactivity of organisms to stress, differences in parent investment lead to different stress reactivity patterns in men and women. Men are more likely to choose “fight or flight” reactivity, while women are more likely to choose the “tend-and-befriend” stress reactivity pattern [47]. This is because women are generally more involved in caring for future generations. Pregnancy, and women's greater investment in caring for their offspring, result in women playing a more important role in the growth of their children. High maternal investment prevents women's stress reactivity from negatively affecting their own and their children's health, and ensures the highest chance of survival for themselves and their offspring. “Taking care” means taking care of offspring and integrating into the surrounding environment, which is very effective in dealing with many kinds of threat. In contrast, the stress reactivity of fighting puts women and their offspring in dangerous situations, and the reactivity of fleeing is not feasible for women who have to conceive and care for their unborn offspring.“Women with PMS/PMDD, due to heightened stress reactivity, may experience difficulties in using adaptive “tend-and-befriend” strategies. Their stress-induced emotional reactivity can make it harder to maintain stable social bonds, which in turn can exacerbate their symptoms, creating a vicious cycle [44].”

Alternative behavioral reactivity may evolve in women. Protecting themselves and future generations in the face of potential threats is a complex and difficult task. Compared to those who cannot use social groups effectively, those who can are more likely to successfully deal with threats. This leads to the hypothesis that women will selectively become sociable in the face of stress, as this will maximize the use of group members as a form of protection for themselves and their offspring. Talyor believes that women's stress reactivity will cause them to take care of their offspring and be close to social groups, which facilitates the process of “becoming friends”, that is, establishing a connected social network in order to obtain more resources for protection from stress [44]. In short, “tend and befriend” is a model for women to cope with stress. “Tend” involves parenting activities aimed at protecting themselves and future generations, increasing safety and reducing irresponsible parenting, while “befriend” is the creation and maintenance of a social support system that facilitates this process. The physiological-behavioral mechanism of the “tend-and-befriend” model seems to mobilize the attachment-care system [48].

Specifically, in stressful situations, women follow the behavioral pattern that provides immediate (tend component) care and protection for future generations, and seek belonging and social support (befriend) in the ideal, same-sex group. This type of stress reactivity shows that when women face stress, their first reactivity is to build and invoke social systems to reduce the impact of stress. Therefore, under stress, women preferentially activate the HPA axis and the oxytocin system [49]. In accordance with the “tend-and-befriend” model, Geary and Flinn proposed a modified model, in which both men and women show desire and demand for social groups after stress experiences [50]. However, the gender differences in biobehavioral stress reactivity will depend on the different types of stressors. Specifically, women are more sensitive to care-related environmental cues, such as baby crying, while men are more sensitive to what they consider to be threats from outside groups. Both men and women show HPA axis and SNS activity in reactivity to physiological stress. However, women's neural network patterns buffer physiological reactivity to stress. The “fight or flight” reactivity mobilizes resources to enhance concentration and alertness and to suppress individual needs in the face of stress. The neural biological reactivity of women under stress occurs in the reward system, and then the “fight or flight” reactivity is adjusted downward [51]. Therefore, women's acute stress reactivity is less pronounced, and women are less susceptible to the deleterious effects of acute stress.

The diathesis-stress model also provides an approach for understanding the role of interactions between genes and the environment in psychological disorders [52]. The diathesis-stress model is a psychological theory designed to explain behavior as a predisposition to susceptibility associated with the experience of stressful life events. The term diathesis is derived from Greek and means tendency or susceptibility, expressed in the form of genetic, psychological, biological, or environmental factors. Individual differences in the population are largely due to differences in their susceptibility to developing or suffering from disease. Diathesis or predisposition interacts with subsequent stress reactivity. Stress refers to a life event or a series of events that can disrupt the psychological balance of individuals, and potentially serve as a catalyst for developing disease [53]. The diathesis-stress model reveals the hereditary characteristics of an individual's susceptibility to specific conditions such as depression, anxiety, or addiction. For a particularly sensitive person, even a few environmental stresses will inevitably cause some kind of mental illness. The diathesis-stress model also shows that genes are only one aspect of the inducement of mental illness, and environmental factors are also important [54].

Specifically, this theory demonstrates that individuals’ biological vulnerability or predisposition to a specific mental illness can be triggered by stressful life events. If someone is resilient or less biologically vulnerable to a disease, a very high level of stress is needed before symptoms of the disease are induced. On the other hand, if a person has a high biological vulnerability to the disease, even lower levels of stress can induce the symptoms of the disease. Before stress levels reach pivotal thresholds, individuals are normally functional, and biological vulnerability is not shown [55]. Therefore, the diathesis-stress model helps to explain why some, but not all, people suffer from mental illness, even though they face the same environmental stressors [56].

In addition, there is a reciprocal relationship between stress and mental illness. Stressful life events can aggravate disease, which in turn, can cause life to be more intense and stressful. Environmental stress can be divided into acute or chronic stress, and can include neglect or abuse by a parent, death of a family member or friend, relationship or marriage issues, or witnessing a traumatic event. One stressful life event is not enough to induce mental illness, but when negative events accumulate, vulnerable individuals may suffer from illness [57]. Early diathesis-stress models focused on pathological diseases such as schizophrenia, depression, and anxiety. Research shows that these diseases tend to be genetic, but also have a significant relationship with stressful life events [58]. Recently, the basic content of the diathesis-stress model has been expanded to include a predisposition to protection of individuals from stress-related disorders, or resilience, rather than focusing on who might be a victim when facing extreme stress. Here, resilience is not the opposite of diathesis. On the contrary, individuals may have overall plasticity differences in negative (e. g., stress) or positive (e. g., supportive) environmental impacts [[59], [60], [61]].

## 3 Stress dysfunction and premenstrual syndrome

### 3.1 PMS shares the equivalently neuroendocrine metabolic circuit based on hormone fluctuations with stress reactivity systems

**Fig. 2:**
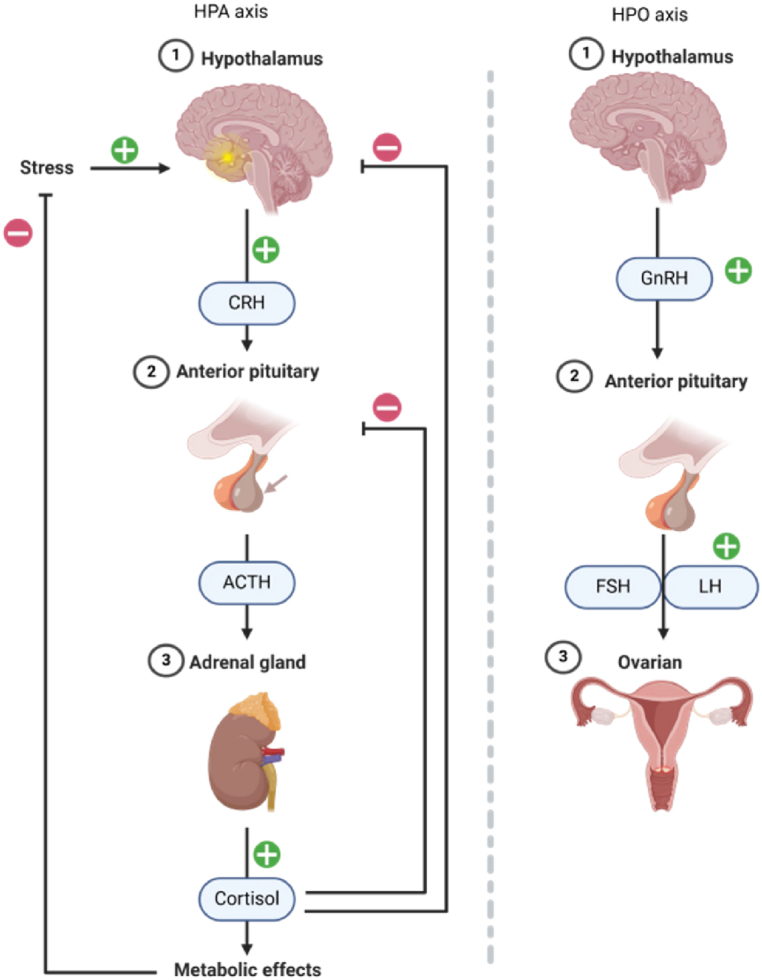
Interaction between the HPA and HPO axes under stress.

Stress can increase premenstrual symptoms, and women with PMS are more prone to nervousness, anxiety, and restlessness [62]. This indicates that there may be a connection between PMS and stress. PMS is mainly caused by sensitivity to hormone fluctuations, and the effect of stress on behavior and physiology is also the result of stress hormone release. Therefore, can the connection between PMS and stress be established from the perspective of hormone fluctuations? In our view, this is possible. “As shown in Fig. 2 below”In the face of stressors, cortisol activation or perception, causes the hypothalamus to release corticotropin-releasing hormone (CRH) and the pituitary gland releases ACTH. Finally, the adrenal gland releases cortisol. The adrenotropin-releasing hormone inhibitor (CRH inhibits GnRH pulse generator) causes the hypothalamus to release gonadotropin releasing factor (GnRH), and then causes the pituitary gland to release luteinizing hormone (LH) or follicle stimulating hormone (FSH). The ovaries then releases estrogen or progesterone, and these hormones finally act on the uterus. Cortisol inhibits the release of progesterone secreted by the pituitary gland, and at the same time inhibits the release of estrogen secreted by the ovaries. Eventually, cortisol will cause the target tissue, namely in the uterus, to resist estrogen [7].“It is evident that hormonal fluctuations play a crucial role in linking PMS and stress. Stress disrupts the hypothalamic-pituitary-ovarian (HPO) axis, leading to menstrual cycle disturbances [63]. Hormonal fluctuations during the cycle, particularly in estrogen and progesterone, not only trigger emotional changes but also heighten sensitivity to stress [64]. In the luteal phase, when PMS symptoms peak, fluctuations in these hormones exacerbate emotional instability and stress responses [65]. Women with PMS are especially sensitive to these changes, creating a negative feedback loop that worsens symptoms. Thus, the menstrual cycle mediates the relationship between stress and PMS through hormonal fluctuations.”

Due to hormone fluctuations, women with PMS/PMDD are more sensitive to negative emotional reactions. The fluctuations in progesterone and estrogen caused by the MC can regulate a woman's emotional symptoms through their functions in the CNS [66].“Our findings demonstrate a significant correlation between elevated cortisol levels during the luteal phase and increased emotional dysregulation in women with PMS. This suggests that hormonal fluctuations, particularly in estrogen and progesterone, exacerbate stress reactivity through HPA axis dysregulation, contributing to the worsening of PMS symptoms [67,68].”

In a normal MC, natural fluctuations of estrogen and progesterone have been shown to cause changes in the central nervous system's gamma-aminobutyric acid (GABA), serotonin, and opioid neurotransmitter responses. Changes in the level of endogenous tetrahydroprogesterone are closely related to dysphoric disorders during the premenstrual and menopausal periods. Research has focused on the progesterone metabolite tetrahydroprogesterone and found that compared to healthy women, aminobutyric acid neurotransmitters have a lower secretion concentration in PMDD women [69]. Insufficient concentrations of this metabolite can lead to increased levels of anxiety, especially when faced with stressful situations. Studies on tetrahydroprogesterone, stress, and related disorders have also shown that changes in the level of tetrahydroprogesterone in response to acute stressors tend to restore the homeostasis of the organism by inhibiting hyperactive hypothalamic-pituitary-adrenal (HPA) axis activity. However, chronic stressors can lead to neuropsychiatric disorders, including depression and anxiety, and this is associated with a decline in tetrahydroprogesterone levels [70].

The above is a comparison of the hormone connection between stress and PMS from the perspective of the downward pathway of hormone secretion. The upward pathway of CNS activity can also establish a correlation between stress and PMS. From the stress nerve response loop, the stress response mainly involves the prefrontal lobe, hippocampus, and amygdala [71]. The amygdala is also the main area of sex hormone expression [72]. In the case of sex hormone fluctuations, the shape and function of the amygdala undergo certain changes [73,74]. While the shape and function of the amygdala are regulated by sex hormones, as a nerve area involved in stress response, the amygdala participates in the process of threat and stress detection. The amygdala will perceive the stress stimulus in the environment and act on it. Emotional judgment activates the neural circuit to project nerve impulses to the area related to the “fight or flight” response, which wakes up the underlying stress response system, including the HPA axis and the ANS [75,76]. Because PMS women are sensitive to gonadal hormone fluctuations, and the amygdala is the main expression area of gonadal hormones, activation of the amygdala involves the central mechanism of stress response and can awaken the underlying pathway of stress response.

Liu and colleagues used PMS women and healthy women as subjects, and combined functional magnetic resonance imaging (fMRI) techniques to explore the alterations of resting-state functional connectivity in the default network in women with PMS. Their results show that compared to healthy women, women with PMS have reduced functional connectivity in the frontal and lateral hippocampus, increased functional connectivity in the left medial temporal superior/temporal gyrus, and the central anterior gyrus. In addition, women with PMS have higher anxiety and depression scores and lower stress perception scores. Finally, for women with PMS, there is a positive correlation between the subjective stress perception score and functional connectivity of the frontal gyrus and wedge-shaped lobe. There was also a negative correlation between depression scores and functional connectivity in the frontal gyrus and anterior wedge, and a positive correlation with functional connectivity in the medial temporal gyrus [77]. Therefore, PMS and hormone fluctuations may share the equivalently neuroendocrine metabolic circuit with the stress reactivity system.

Recent neuroimaging studies utilizing fMRI, EEG, and NIRS have significantly deepened our understanding of PMS and its relationship with brain function. Liu and colleagues identified notable disruptions in functional connectivity within the precentral gyrus and thalamus, which play key roles in emotional regulation and cognitive control [78]. Similarly, Duan and colleagues found altered connectivity between the hippocampus and middle cingulate cortex (MCC), suggesting that PMS patients experience abnormal sensory and emotional processing [79]. Aoki et al. used NIRS to reveal reduced activation in the prefrontal cortex during the luteal phase, which was associated with increased emotional dysregulation and cognitive challenges [80]. Moreover, Liu et al. highlighted frequency-specific changes in the striatum and thalamus, especially in the slow-5 band, suggesting these may serve as diagnostic biomarkers for PMS [81]. Additionally, Gao et al. found that women with PMDD exhibit heightened frontal lobe activity in response to stress, indicating an intensified neural reaction to emotional stimuli compared to healthy controls [82].

These studies collectively suggest that PMS and stress reactivity share common neuroendocrine pathways, with frequency-specific alterations in brain regions such as the striatum, thalamus, and prefrontal cortex. Hormonal fluctuations during the menstrual cycle significantly impact brain function, particularly in areas involved in emotional regulation and cognitive control, further emphasizing the complex relationship between PMS, brain connectivity, and stress response mechanisms.

### 3.2 There is a comorbidity between PMS and stress-related disorders

Compared to men, women have a significantly higher risk of developing PTSD, which causes a social and psychological burden on women and increases their medical expenses [83]. Studies have shown that changes in hormones during the MC may have an impact on women suffering from PTSD. The evidence supporting this hypothesis is that premenstrual disorders and PTSD share some similar clinical features, such as irritability, anxiety, and sleep disturbances [24]. In addition, some studies have also found that patients with premenstrual disorder and PTSD have ANS dysfunction. For example, patients with PTSD and premenstrual disorder have low levels of HRV, which is a manifestation of resting physiological overreaction [84]. In other words, autonomic dysfunction may partly reflect a certain mechanism shared by PTSD and premenstrual disorders. Some epidemiological studies have also shown a link between moderate to severe PMS/PMDD and PTSD [[85], [86], [87]].

Studies have also shown that women with PTSD are more likely to develop severe PMS or PMDD [83]. McKinnon conducted a study on the relationship between traumatic experience and PMS in female soldiers and found that among female soldiers, trauma history, especially sexual trauma, is associated with moderate to severe PMS [88]. Other studies have also confirmed that there is a relationship between trauma history and PMS [89]. A series of studies have shown that compared to women who have not been abused, women who self-reported sexual abuse are more likely to develop PMS/PMDD, and they also have more severe premenstrual symptoms [90]. Studies including neurobiological indicators have shown long-term exposure to an abusive environment causes continuous dysfunction of the stress reactivity, which supports the hypothesis that trauma and stress may be partly related to the pathophysiology of PMS/PMDD [91].

A community-based study of 568 women between the ages of 18 and 45 found that there is a significant correlation between adolescent physical and sexual abuse experiences and PMS [28]. Perkonigg et al. [86] and Wittchen et al. [87] used the same batch of data from a sample of German women (1251, with a base age between 14 and 24, a batch of tracking data over 4 years). The results showed that, independent of age, there was a significant correlation between the basic PTSD value and the incidence of PMDD. Pilver et al. [86] explored the relationship between premenstrual symptoms and PTSD from the perspective of female traumatic experience and found that trauma and PTSD are both related to PMS and PMDD, and the relationship is independent. Takeda et al. [29] also found significant comorbidity between PMS/PMDD and PTSD in a sample of Japanese women who experienced major earthquake disasters. However, this evidence is not sufficient to prove that PMS or PMDD is a type of stress dysfunction, or that its pathology is related to stress. Therefore, further research is necessary to conduct an in-depth exploration into the combination and comparison of stress neural mechanisms and PMS female neural activity.

### 3.3 PMS itself may act as a stressor and potentially create a negative feedback loop to worsen symptoms

For women, the premenstrual period itself is a source of stress, so women's stress reactivity will affect the severity of their premenstrual symptoms [92]. Self-awareness of stress is one of the risk factors for PMS [93]. There is evidence that compared to healthy women, women with PMS/PMDD will subjectively report feeling greater stress [30]. The evidence for this view comes from experiments that compare the cardiovascular responses of women with different levels of symptoms to physical/cognitive stressors [94]. Specifically, women with PMDD report experiencing more stressful life events, and the stressor itself seems to have a greater impact on their lives [95]. Furthermore, in the face of various stressors, women with PMDD seem to have a significantly slower heart rate and diastolic blood pressure response, and they also have a slower heart rate and cardiovascular output response. Importantly, these sluggish cardiovascular responses are independent of their menstrual phases when women undertaking the experiment [96]. Liu and colleagues conducted a brain electrical stress assessment test and a physiological stress assessment test on women with PMS and healthy controls to investigate the stress reactivity variations of PMS and found that women with PMS have persistently or permanently abnormal emotions and stress reactivity that is independent of MC compared to healthy women [97]. Therefore, as a potential source of stress, PMS itself will affect the stress reactivity of women with PMS. The pathophysiological basis of PMS is partly related to the neural mechanism of stress reactivity.

### 3.4 The application of biofeedback training of stress disorder is suitable for PMS treatment

When considering the basis for intervention and treatment of PMS, the fact that the target women are of childbearing age and in the reproductive period must be taken into account. Therefore, hormone, drug treatments and taking oral contraceptives are not ideal. Biofeedback is a noninvasive, relatively safe, and effective way to allow individuals to perceive and control their own bodies and make individuals more in control of their own health [98]. “Biofeedback is a noninvasive, relatively safe, and effective way to allow individuals to perceive and control their own bodies and make individuals more in control of their own health. Considering the heightened stress response in PMS patients, biofeedback techniques aimed at regulating the sympathetic nervous system and heart rate variability (HRV) could serve as effective interventions. Recent studies have shown that HRV biofeedback not only alleviates the physiological symptoms of stress but also enhances emotional resilience, thereby reducing PMS-related stress and its associated symptoms [99,100]”The benefits of biofeedback include improved moods, less negative emotions, enhanced task performance, improved response to stressful situations and improved immune function. Electroencephalography (EEG) biofeedback is real-time feedback to EEG variables, that is, those variables that are sensitive to arousal status to help regulate arousal. After biofeedback training, the organism will experience a physiological state of calm, which can then allow the individual to feel their ability to control and regulate the body. This calm state after physiological adjustment is consolidated into the brain [101]. The changes that the individual can experience afterwards include reduced anxiety, stable heart rate, adjusted body temperature, reduced panic, reduced anger, and less restlessness. EEG biofeedback has been proven to effectively regulate the body and improve physical symptoms. With the help of biofeedback, emotional stress can be reduced or eliminated [102].

The main manifestation of PMS is emotional and physiological dysregulation, and the purpose of biofeedback is to re-regulate, thus biofeedback is a useful tool for regulating PMS. Using biofeedback, the training of the abnormal stress reactivity pattern improves the stress disorder symptoms of women with PMS. The result of the improvement is that the ability of PMS women to cope with stress has improved. Specifically, biofeedback has successfully confirmed its effect on stress management, and it also has a good improvement effect on stress-related disorders [[103], [104], [105], [106], [107]]. The improvement of biofeedback training for women with PMS is that training improves the underlying mechanism rather than the specific external symptoms. The symptoms of PMS appear in situations of insufficient or excessive arousal, which indicates that women with PMS may have dysfunction in their overall state [108,109]. Low mood and insufficient autonomic nervous response are signs of insufficient PMS arousal, while body swelling and pain can be considered signs of excessive PMS arousal [110,111]. With the help of EEG biofeedback, researchers can enhance the arousal of the cortex, and further enhance the cortical control ability and physiological self-regulation ability, with the ultimate goal of improving the dysfunctional state. For example, Liu et al. [112] used biofeedback equipment to conduct 20-day stress reactivity improvement training for women with PMS to explore the neural plasticity of stress dysfunction in women with PMS. Their results indicate that biofeedback training can improve the stress dysfunction of women with PMS, specifically by increasing the frontal EEG lateralization score and reducing negative emotions.

## 4 Conclusion

Above all, we consider the PMS as a type of stress dysfunction with evidences from four aspects: namely 1) PMS shares the equivalently neuroendocrine metabolic circuit based on hormone fluctuations with stress reactivity systems; 2) There is a comorbidity between PMS and stress-related disorders; 3) PMS itself may act as a stressor and potentially create a negative feedback loop to worsen symptoms; and 4) The application of biofeedback training of stress disorder is suitable for PMS treatment. These indicate that the neural mechanism that depends on the stress activity changes caused by hormone fluctuations during the menstrual cycle has been partly identified.

## Generative AI statement

Generative AI tools were used for brief language polishing in the writing process, with all content reviewed and finalized by the authors.

## CRediT authorship contribution statement

**Qing Liu:** Writing – review & editing, Writing – original draft, Visualization, Validation, Supervision, Resources, Project administration, Methodology, Investigation, Funding acquisition, Formal analysis, Data curation, Conceptualization. **Yuhang Lin:** Writing – review & editing, Methodology, Investigation. **Wenjuan Zhang:** Writing – review & editing, Formal analysis, Conceptualization.

## Availability of data and materials

All data, models, and codes generated or used during the study appear in the submitted article.

## Funding statement

This study received no specific funding.

## Declaration of competing interest

The authors declare no conflicts of interest.

## Abbreviations

MC: menstrual cycle
PMS: premenstrual syndrome
PMDD: premenstrual dysphoric disorder
DSM-V: the fifth edition of the diagnostic and statistical manual of mental disorders
GAD: generalized stress disorder
SNS: sympathetic nervous system
ANS: autonomic nervous system
HPA: hypothalamic-pituitary-adrenal
HR: heart rate
BP: blood pressure
CRF: corticotrophin releasing factor
ACTH: adrenocorticotrophic hormone
CNS: central nervous system
PTSD: post-traumatic stress disorder
CRH: corticotropin-releasing hormone
GnRH: gonadotropin releasing factor
LH: luteinizing hormone
FSH: follicle stimulating hormone
GABA: gamma-aminobutyric acid
fMRI: functional magnetic resonance imaging
EEG: electroencephalography
